# Chirality Probe of Twisted Bilayer Graphene in the Linear Transport Regime

**DOI:** 10.1021/acs.nanolett.4c00371

**Published:** 2024-04-08

**Authors:** Dario A. Bahamon, Guillermo Gómez-Santos, Dmitri K. Efetov, Tobias Stauber

**Affiliations:** † School of Engineering, Mackenzie Presbyterian University, São Paulo 01302-907, Brazil; ‡ MackGraphe Graphene and Nanomaterials Research Institute, Mackenzie Presbyterian University, São Paulo 01302-907, Brazil; § Departamento de Teoría y Simulación de Materiales, Instituto de Ciencias de Materiales de Madrid, CSIC, E-28049 Madrid, Spain; ∥ Departamento de Física de la Materia Condensada, Instituto Nicolás Cabrera and Condensed Matter Physics Center (IFIMAC), Universidad Autónoma de Madrid, E-28049 Madrid, Spain; ⊥ Fakultät für Physik, Ludwig-Maximilians-Universität, Schellingstrasse 4, D-80799 München, Germany; # Munich Center for Quantum Science and Technology (MCQST), Schellingstrasse 4, D-80799 München, Germany

**Keywords:** Quantum Transport, Chirality, Twisted Bilayer Graphene, Reciprocity Relations

## Abstract

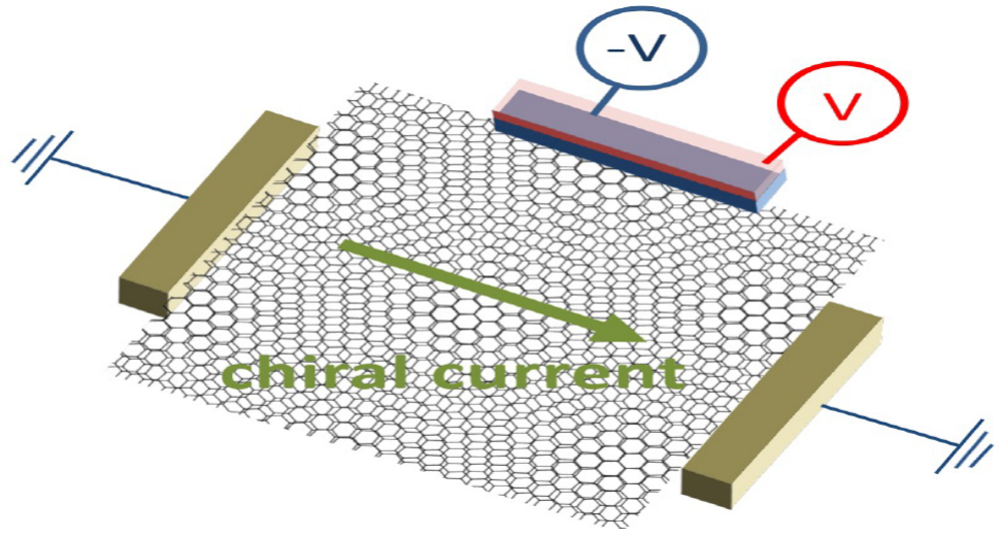

We propose minimal transport experiments in the coherent regime that can probe the chirality of twisted moiré structures. We show that only with a third contact and in the presence of an in-plane magnetic field (or another time-reversal symmetry breaking effect) a chiral system may display nonreciprocal transport in the linear regime. We then propose to use the third lead as a voltage probe and show that opposite enantiomers give rise to different voltage drops on the third lead. Additionally, in the scenario of layer-discriminating contacts, the third lead can serve as a current probe capable of detecting different handedness even in the absence of a magnetic field. In a complementary configuration, applying opposite voltages on the two layers of the third lead gives rise to a chiral (super)current in the absence of a source–drain voltage whose direction is determined by its chirality.

The idea of chirality permeates many branches of science,^1−3^ and also in condensed matter physics the concept is used to describe electronic properties in reciprocal and real space. In reciprocal space, chirality defines the handedness of the spin of the electron to its momentum,^4−6^ and in real space, chirality depicts the handedness of molecules and solids that cannot be superimposed onto their mirror images.^1,7^ Most generally, chirality always emerges when discrete symmetries such as reflection, time-reversal, or particle-hole symmetry are broken.

Independent of the setting, chiral systems offer opportunities to observe new phenomena as well as challenges regarding their detection.^6−8^ The signature of topological insulators, e.g., is the existence of chiral dissipationless states at the boundaries of the sample,^4,6,8^ and the electrical detection of these states requires nonlocal transport measurements.^9^ On the real space side, chiral molecules spin polarize the electric current passing through them,^10^ and the chiral-induced spin selectivity (CISS) effect is detected in a two-terminal configuration as a nonlinear *I*–*V* characteristic.^7^ Also, electrical magneto-chiral anisotropy in a classical four-terminal configuration has been observed, where the longitudinal enantioselective magnetoresistance is nonlinear in the current.^11−13^ Finally, in noncentrosymmetric materials, chirality manifests itself in various nonreciprocal response phenomena^14−18^ where the resistance depends linearly on the current, causing a nonlinear voltage drop in a two-terminal setup.^19−21^

van der Waals moiré materials offer new opportunities to engineer certain geometric structures that can lead to novel properties.^21−23^ For example, twisted bilayer graphene^24−29^ (TBG), which has recently attracted great attention due to the discovery of novel electronic phases,^30−34^ can be rotated clock- or anticlockwise. Due to the finite interlayer separation, TBG heterostructures with opposite twist angles can only be superimposed onto each other after performing a mirror reflection with respect to the *xy*-plane. Thus, TBG is intrinsically chiral which has been experimentally demonstrated by observing optical dichroism without breaking time-reversal symmetry.^35^ The effect becomes largest for frequencies which induce transitions between states that are maximally delocalized between the two layers such that the misalignment between layers is most effective,^36,37^ but also in the dc-limit, the chirality of TBG is manifested by intrinsic magnetic–electric coupling.^38−51^ In the dc-limit with broken time reversal symmetry, an electrical magnetochiral anisotropy is anticipated to emerge.^52^ Nevertheless, in conventional transport experiments where the net current in voltage leads is maintained at zero,^53,54^ the influence of chirality has not been a significant factor thus far.

In this work, we precisely fill in this gap by investigating electronic transport through TBG in the linear regime within the Landauer–Büttiker formalism. Using general symmetry arguments, we point out that in order to distinguish different enantiomers, i.e., samples with twist angles θ and −θ, respectively, it is crucial to have three leads. This is contrary to the zero-field superconducting diode effect where two leads are sufficient.^16,55−61^ The third lead can now be used as a voltage probe that detects the chirality if a magnetic field is applied parallel to the layer. Or, in the case of layer-discriminating contacts, it can be used as a current probe even in the absence of a magnetic field. We thus show that it is not necessary to break time-reversal symmetry with a magnetic field in order to observe the layer-discriminating transverse current effect in a typical transport experiment.

## Landauer Formalism and General Symmetries

We consider two systems with opposite twist angles, ±θ or chiralities, related to each other by a mirror symmetry with respect to a horizontal plane midway between the layers. Notice that in this symmetry operation we include the leads, which we consider, for the moment, equally coupled to both layers. The reason for this layer-symmetric attachment of leads is 2-fold: first, hopefully easier experimental realization, and second, simplified chiral analysis for, otherwise, a right lead attached to the bottom layer in the θ flake would become a right lead, but in the top layer for the mirror flake with opposite chirality −θ. Let us also assume that there are *N* leads attached to the system and that the current *I*_*p*_ in lead *p* is^54^
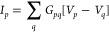
1where *V*_*q*_ is the potential of lead *q* over ground level, and the conductance  is proportional to the total transmission *T̅*_*pq*_ between lead *q* and lead *p*. Because of the inherent symmetries of the transmission matrices, the following reciprocity relation holds^54^
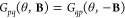
2where **B** is the magnetic field (see the Supporting Information (SI)). In our case, it denotes an in-plane magnetic field.

**Figure 1:**
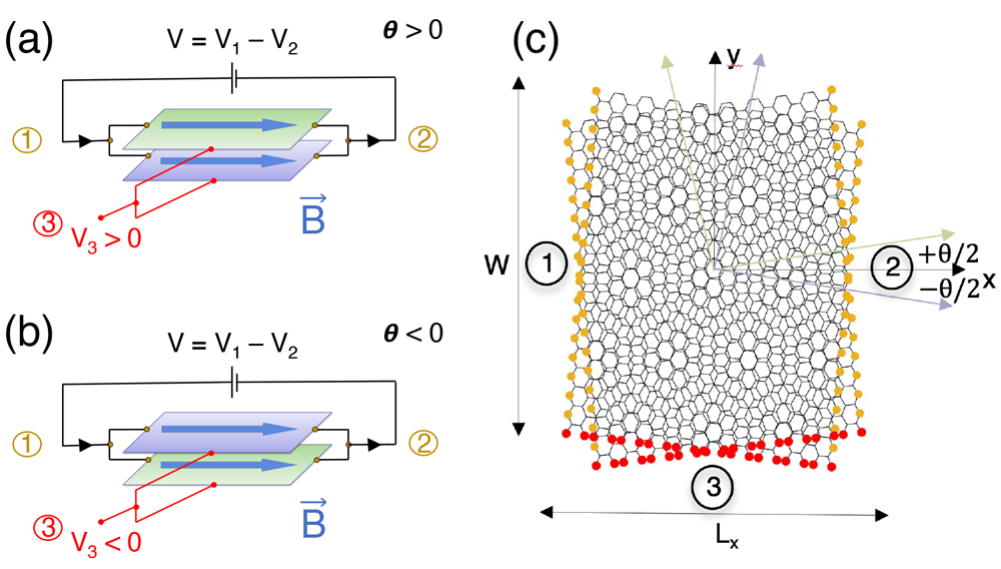
(a) and (b) Proposed setup for voltage probe detection of chirality in the presence of a magnetic field (**B** = *B***e**_*x*_). The reading of voltage *V*_3_ is opposite for the opposite chiralities depicted. The green color stands for a layer twisted by a positive angle, while the violet means a negative twisted angle layer. (c) Schematic representation of the twisted bilayer graphene (TBG) three-terminal device, and the yellow and red dots represent the atomic sites in contact with leads 1, 2, and 3, respectively.

We now perform a *z*-reflection by mapping *z* to −*z* for each piece of matter, including sources if external fields are present. This is the composition of space inversion, (*x*, *y*, *z*) → (−*x*, −*y*, −*z*), followed by a π-rotation around the *z*-axis (see Figure 1(c)), where the origin is placed in the center and midway between the planes. By this, one maps θ → −θ, *p* → *p*, *q* → *q*, and, crucially important here, **B** → −**B**; i.e., **B** is an axial vector, first unchanged by space inversion but later changing sign upon the π-rotation around the *z*-axis. In twisted systems, we thus also have the *chiral* reciprocity relation
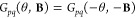
3valid for in-plane magnetic fields and layer-symmetric attachments. This correlation shows that there is no way to detect the sign of the chirality without magnetic fields in twisted arrangements with layer-symmetric leads because *G*_*pq*_(θ, **B** = 0) = *G*_*pq*_(−θ, **B** = 0). On the other hand, the correspondence expressed in eq 3 implies a locking of chirality and field.

To exploit this relation, let us now fix the direction, **e**_*n*_, of the in-plane magnetic field, **B** = *B***e**_*n*_ and expand the conductance to linear order in *B*
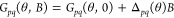
4
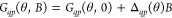
5In conjunction with eqs 2 and 3 and knowing, as stated earlier, that *G*_*pq*_(θ, 0) = *G*_*qp*_(θ, 0) = *G*_*qp*_(−θ, 0), we arrive at
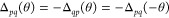
6The above equation indicates an electrical mechanism to detect the chirality of twisted devices through conductance measurements even with layer-symmetric leads.

## Including the Third Lead As a Voltage Probe for Chirality

Let us now focus on the minimal system that can detect chiral properties in the linear regime. The device with three leads is depicted in Figure 1(a,b). All leads couple equally to both layers, as previously assumed, and leads 1 and 2 have an equal number of channels. In the absence of a magnetic field, the currents can be obtained from eq 1
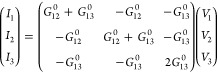
7where it is defined that *G*_*pq*_(±θ, **B** = 0) = *G*_*pq*_^0^ = *G*_*qp*_^0^ as well as assumed, based on the symmetry of the problem, that *G*_13_^0^ = *G*_23_^0^. When **B** ≠ 0, the additional contribution to the conductance matrix is
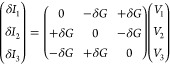
8where the corrections are characterized by a single parameter to linear order in |**B**|, δ*G* = Δ(θ)*B*. This is a consequence of the conductance sum rule ∑_*q*≠*p*_*G*_*pq*_(+**B**) = ∑_*q*≠*p*_*G*_*pq*_(−**B**), which implies that ∑_*q*≠*p*_ Δ_*pq*_ = 0. Now, we can use the third lead as a voltage probe, imposing *I*_3_ = 0 while applying a source–drain voltage drop, *V*_1_ = *V*/2 and *V*_2_ = −*V*/2. Then to linear order in the *B*-field, the voltage probe, *V*_3_, yields
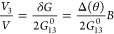
9Previously, it was demonstrated that Δ(θ) = −Δ(−θ), which ensures that the voltage probe becomes also a probe of the chirality sign (*V*_3_(θ) = −*V*_3_(−θ)).

### Numerical Implementation

Having established the basic relations that allow the detection of handedness through electric measurements, our attention turns to their numerical calculation in a TBG region of dimensions *W* × *L*_*x*_, where both *W* and *L*_*x*_ are set to 50 nm (the system is sketched in Figure 1(e)). The computation is performed employing the tight-binding model and Green’s functions. It is crucial to note that we opt for multiple neighbors for each site to replicate the characteristics of TBG. As a consequence, the charge neutrality point (CNP) emerges at approximately *E* ≈ 0.2961*t*_0_, where *t*_0_ represents the nearest neighbor hopping. Consequently, all numerical outcomes presented in this study are offset by this value and *E*_*F*_ = *E* – 0.2961*t*_0_. For detail, we refer to the Supporting Information. There, we also show that the reciprocity relations, as delineated in eqs 2 and (3) are correctly implemented in our numerical calculations.

## Detecting Chirality by Voltage Probe with a Magnetic Field

**Figure 2:**
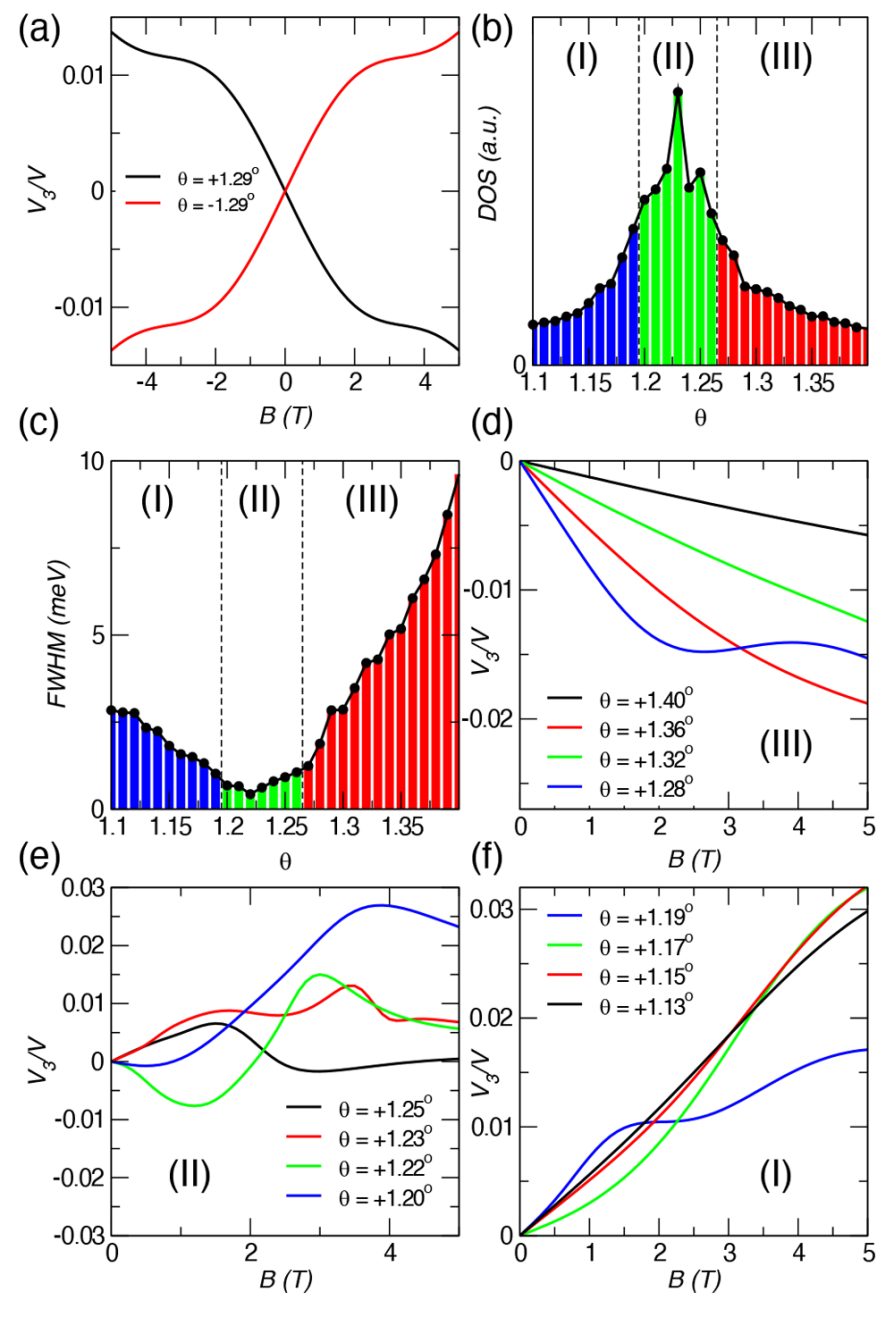
(a), (d–f) *V*_3_ for different twist angles as a function of **B** parallel to the *x*-axis. (b) Value and (c) full width at half-maximum (fwhm) of the DOS peak without a magnetic field. The Roman numerals I, II, and III, along with the shaded regions, serve as visual guides to differentiate various chirality behaviors. In the panels where *V*_3_/*V* is presented, the Fermi energy is set to zero, and *E*_F_ = 0.

We now consider the case where *V*_1_ = *V*/2, *V*_2_ = −*V*/2, and *I*_3_ = 0. Using this condition, *V*_3_/*V* = (*G*_31_ – *G*_32_)/(2*G*_31_ + 2*G*_32_) can be calculated as a function of the magnetic field for different twist angles and energies. For θ = ±1.29° and Fermi energy *E*_F_ = 0 meV, Figure 2(a) shows that *V*_3_/*V* is linear in *B* with opposite slope for opposite twist angles, confirming the prediction of eq 9 that lead 3 also becomes a probe for chirality and that quantum transport is sensitive to chirality. However, it is not possible to relate in advance the sign nor the strength of the linear field dependence to a certain twist angle. This is similar to previous observations for the infinite system,^37,38,40^ where the chiral component of the conductivity was shown to exhibit highly nonmonotonous behavior with filling factor and twist angle.

For large angles, the effect of the handedness of the junction on transport is negligible, and *V*_3_/*V*_1_ ≈ 0. This already indicates that the effect is mainly electronically and not configurationally driven, for both layers that are highly decoupled for large angles. For small angles, the density of states (DOS) is presented in Figure 2(b–c) in terms of the largest value as well as the full width at half-maximum (fwhm) of the main peak. From there, we infer that the magic angle is located around ∼1.23° where the highest and narrowest DOS peak appears and serves to diagnose the magic-angle regime.

Additionally, three regions are shaded and identified by the Roman numbers I, II, and III conforming to the behavior of *V*_3_. For θ > 1.26° (region III in red), a linear-in-*B* regime is perfectly defined with negative slopes for positive twist angles as shown in Figure 2(d). In the blue shaded region I (θ < 1.20°), the signal of the linear response is inverted, and positive slopes appear for positive twist angles; characteristic lineshapes of *V*_3_ for angles in these region are presented in Figure 2(f).

Around the magic angle, the linear relation on the magnetic field becomes weaker, and the nonlinear *B*-dependence dominates the response as shown in Figure 2(e). However, even for twist angles in this region, the chirality obeys the symmetry relation *V*_3_(+θ) = −*V*_3_(−θ), as discussed in the SI, where also the effects of different coupling strengths to lead 3 are presented.

Let us finally emphasize that our approach utilizes the Landauer–Büttiker formalism within the linear regime. In this regime, the conductance *G* is computed in equilibrium, thus avoiding any dependence on the current density. This distinction ensures that in our setup the relationship between the voltage probe reading, *V*_3_, and the source–drain voltage (and current) remains linear.

## Detecting Chirality by a Current Probe without a Magnetic Field

Let us now discuss how to detect the chirality of a system without a magnetic field. In the infinite TBG, the transverse conductivity is equal in magnitude but of opposite direction with respect to the two layers due to basic symmetry constraints.^37^

### Inducing a Transverse Current

**Figure 3:**
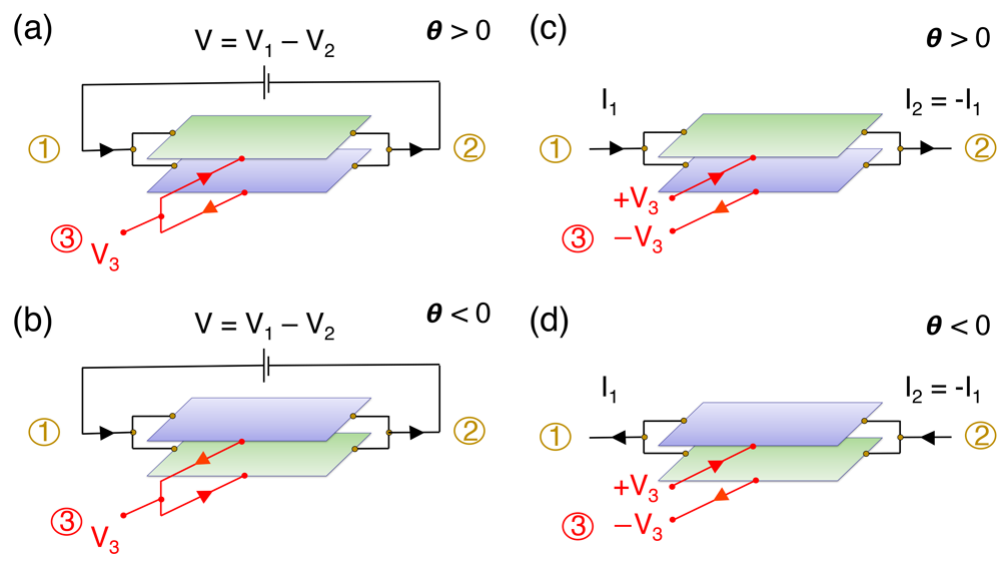
(a and b) Proposed setup for chirality detection by a current probe without a magnetic field. Top and bottom transverse currents reverse direction for the opposite chiralities depicted. The green color stands for a layer twisted by a positive angle, while the violet means a negative twisted angle layer. The detection of chirality by a current probe requires now four leads (1, 2, 3t, and 3b) because the layer index top and bottom of lead 3 is now discriminated. (c) and (d) Alternative setup showing the same electromagnetic coupling without a magnetic field. Now, there is no source–drain voltage; i.e., the potential *V*_1_ and *V*_2_ are equal, but a potential difference between the top and the bottom layer induces a current whose direction depends on the chirality.

In the setup proposed in Figures 3(a,b), we expect to see layer-discriminating transverse currents without the presence of a magnetic field, as they are allowed by the same symmetry principles. This implies that a source–drain voltage *V* = *V*_1_ – *V*_2_ between lead 1 and lead 2 will be accompanied by transverse currents in both layers flowing in opposite directions. As *V*_3*t*_ = *V*_3*b*_, one can interpret this as a vertical “supercurrent” flowing from one layer to the other. In an infinite system, this effect implies that a net current in the *x* direction is accompanied by layer-opposite currents in the *y* direction, which can be thought of as an in-plane magnetic moment along the net flow, a hallmark of chirality.^37,38,62−64^

**Figure 4:**
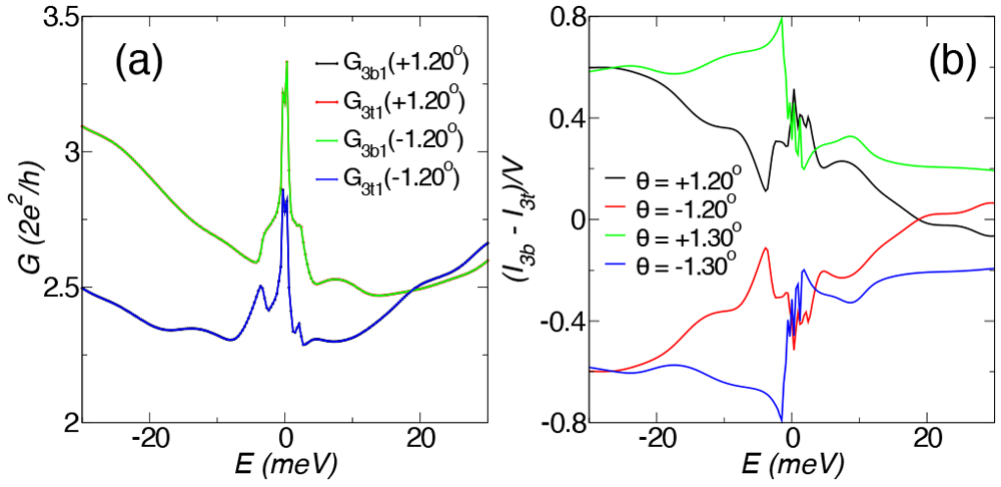
(a) Conductance (in units of 2*e*^2^/*h*) from the lead 1 to lead 3 bottom layer (*G*_3*b*1_) and from the lead 1 to lead 3 top layer (*G*_3*t*1_) for θ = ±1.20°. (b) *G*_3*t*1_ – *G*_3*b*1_ = (*I*_3*b*_ – *I*_3*t*_)/*V* (in units of 2*e*^2^/*h*) for θ = ±1.20°, ±1.30°. From the formalism sign conventions, the finite value of the plotted magnitude implies currents of equal magnitude, |*I*_3*b*_| = |*I*_3*t*_|, running opposite in opposite leads and reversing direction upon chirality reversal, as depicted in Figure 3(a,b).

We need first to generalize eq 3 by including the layer index via *q* → *qν* with ν = *t*, *b*. Performing the reflection and assuming the same coupling between the two layers as schematically shown in Figures 3(a,b), we then have
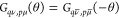
10where ν̅ and μ̅ denote the opposite layer of ν and μ. We can now extend eq 7 to effectively four leads: leads one and two remain layer-symmetric as before, while the original third lead splits into top (3*t*) and bottom (3*b*) (see Figure 1(c,d) and SI for reference). With the above in mind, in Figure 4(a) we show the conductance from lead 1 to lead 3 in the top layer, *G*_3*t*1_, and the conductance from lead 1 to lead 3 in the bottom layer, *G*_3*b*1_. Since the contacts are symmetric, there would be no difference in the two conductances if the system were achiral without a magnetic field. However, a clear difference is seen, giving rise to a chiral current probe in the absence of a magnetic field. Note that reversing the angle maps the conductance *G*_3*b*1_(θ) to *G*_3*t*1_(−θ) and vice versa, as dictated by the general symmetry relations of eq 10. Notice that there is no need for dealing separately with lead 2 in our geometry *G*_3*t*2_ = *G*_3*b*1_ and *G*_3*t*1_ = *G*_3*b*2_.

Now, we can consider the arrangement schematized in Figure 3(a,b), *V*_1_ = *V*/2, *V*_2_ = −*V*/2, and *V*_3*b*_ = *V*_3*t*_ = 0, in which no *net* current flows between the system and the two 3*t* and 3*b* reservoirs: *I*_3*b*_ + *I*_3*t*_ = 0. Yet, a finite current from the upper lead to the lower lead can then be deduced from the finite value of *δG*(θ) = (*I*_3*b*_ – *I*_3*t*_)/(*V*_1_ – *V*_2_) = *G*_3*t*1_(θ) – *G*_3*b*1_(θ). Remember that, from the sign conventions of the formalism, this implies currents of equal magnitude, |*I*_3*b*_| = |*I*_3*t*_|, running in opposite directions in the top and bottom leads, despite the terminals 3*t* and 3*b* having equal potentials, as illustrated in Figure 1(c,d). Reversing the chirality reverses the layer currents, *I*_3*b*(*t*)_(θ) = −*I*_3*b*(*t*)_(−θ); therefore, the experimental detection of these currents becomes a probe for chirality without a magnetic field (*δG*(θ) = −*δG*(−θ)), as shown in Figure 4(b).

The detection of this vertical “supercurrent” can be difficult with real lossy leads. Alternatively, one could use the top and bottom third leads as independent voltage probes, whose different readings would then reveal the chirality, as explained in the SI.

### Inducing a Longitudinal Current

A complementary configuration based on the same symmetry principles is presented in Figure 3(c,d), where the same phenomenon also gives rise to a “supercurrent” from reservoirs 1 to 2 without any voltage drop between them, *V*_1_ = *V*_2_ = 0, when now the transverse currents are driven by a corresponding voltage drop, *V*_3*t*_ = −*V*_3*b*_ = *V*.

A finite current of magnitude |*I*_1_| = *I*_2_| from the lead 1 to lead 2 (or vice versa) can then be deduced from the finite value of *δG*(θ) = (*I*_1_ – *I*_2_)/(*V*_3*t*_ – *V*_3*b*_) = *G*_3*t*1_(θ) – *G*_3*b*1_(θ). Note that *δG*(θ) is given by the same expression as in the previous subsection and is shown in Figure 4(b). The electromagnetic coupling is thus the same in both cases as already discussed in ref (37). Further discussion can be found in the SI, where it is also shown how to instead use terminals 1 and 2 as voltage probes to bypass the difficulties of lossy leads in the “supercurrent” detection.

## Final Remarks

We have proposed two linear transport experiments that can detect the intrinsic handedness of chiral systems. First, exact symmetry arguments were provided to show that a voltage probe can become a chirality probe in the presence of an in-plane magnetic field for external leads that couple symmetrically to both layers. Second, we also demonstrated that a current probe discriminating between the two layers can become a chirality probe even in the absence of a magnetic field. Different enantiomers can thus be distinguished by minimal transport experiments that will hopefully shed more light on this intriguing symmetry, which is also present in various organic molecules.

Using the Landauer–Büttiker formalism, the voltage reading of the third lead and the layer-discriminating, opposite currents between the system and third reservoir(s) were explicitly calculated for a finite sample of twisted bilayer graphene (TBG), confirming our predictions. In both situations, when approaching the magic angle, there is a change of chirality measured through electronic means (voltage and current probes) that does not correspond to an inversion of the actual twist angle between the layers.^40^ In the case of layer-discriminating leads, we showed that in the presence of chirality a net current flow between source and drain reservoirs is accompanied by transverse currents, opposite in each layer. This provides a realization in a Landauer–Büttiker scenario, more accessible experimentally, of predictions previously made^37,38,40^ on the basis of linear response for infinite systems, that in-plane magnetic moments should accompany net current flows in chiral bilayers.

Our one-particle formalism does not allow for symmetry-broken ground states. However, for a time-reversal symmetry (TRS) broken ground state |GS⟩ we expect an alternative *chiral* reciprocity relation even in the *absence* of a magnetic field, involving only the ground state  where  denotes a the antiunitary time-reversal operator. This relation allows for the detection of a TRS broken ground state by measuring a nonzero voltage at the third lead at **B** = 0.

Our discussion should also be relevant for other chiral systems, e.g., for those displaying the planar Hall effect.^65,66^ Then, the continuous variable θ denoting the twist angle is simply replaced by a discrete variable χ = ±1 denoting the different enantiomers. Even the emergence of a magic angle seems to be more general since it can be observed in Weyl semimetals, too.^67^ Let us finally note that the third lead can also be realized by a local probe, especially in the layer-discriminating case. Real-space mapping of the handedness and thus small-angle deviations around the magic angle should be detectable.

## Supporting Information Available

Green’s functions and tight binding method, magic angle regime and density of states, dependence of the voltage probe on magnetic field direction and coupling, reciprocity relations for the two-terminal conductance, relations beyond linear order, Landauer–Büttiker description of the layer-discriminating third lead, and change in chirality in the inifinite system with time-reversal symmetry (PDF)
