# Supplementary material for: Chirality Probe of Twisted Bilayer Graphene in the Linear Transport Regime
Source: Nano Lett. 2024 Apr 8;24(15):4478–84. doi: 10.1021/acs.nanolett.4c00371 (PMC11036400; doi:10.1021/acs.nanolett.4c00371)
Supplement: Supplementary file 1 — nl4c00371_si_001.pdf [file nl4c00371_si_001.pdf]

Supporting Information: Chirality probe of twisted bilayer graphene in the linear transport regime

Dario A. Bahamon,*,†,‡,¶ Guillermo Gómez-Santos,*,§ Dmitri K. Efetov,*,‖,⊥ and Tobias Stauber*,¶

†*School of Engineering, Mackenzie Presbyterian University, São Paulo - 01302-907, Brazil*

‡*MackGraphe Graphene and Nanomaterials Research Institute, Mackenzie Presbyterian University, São Paulo -01302-907, Brazil*

¶*Departamento de Teoría y Simulación de Materiales, Instituto de Ciencias de Materiales de Madrid, CSIC, E-28049, Madrid, Spain*

§*Departamento de Física de la Materia Condensada, Instituto Nicolás Cabrera and Condensed Matter Physics Center (IFIMAC), Universidad Autónoma de Madrid, E-28049 Madrid, Spain*

‖*Fakultät für Physik, Ludwig-Maximilians-Universität, Schellingstrasse 4, D-80799 München, Germany*

⊥*Munich Center for Quantum Science and Technology (MCQST), Schellingstrasse 4, D-80799 München, Germany*

E-mail: dario.bahamon@mackenzie.br; guillermo.gomez@uam.es; dmitri.efetov@lmu.de; tobias.stauber@csic.es

Green´s functions and tight binding method

Employing the Green's functions we have $G_{pq} = \frac{2e^2}{h}\mathrm{Tr}[\Gamma_p \mathcal{G} \Gamma_q \mathcal{G}^\dagger]$, where $\mathcal{G} = [E - H_{TBG} - \Sigma_1 - \Sigma_2 - \Sigma_3]^{-1}$ is the Green's function of the central region, $\Sigma_{1(2)(3)}$ are the self-energies of the leads and $\Gamma_{1(2)(3)} = i[\Sigma_{1(2)(3)} - \Sigma^\dagger_{1(2)(3)}]$ are the couplings of the central region to the leads. Without loss of generality we can assume a wide band model for the leads, that is a constant density of states (DOS) around the Fermi energy, which is typical in metallic contacts. In that case, the self-energy term can be written as $\Sigma_{1(2)(3)} = -i\pi\rho|t|^2$,[S1] were ρ is de DOS of the contact and t is the hopping parameter between the leads and the central twisted region. To guarantee a large number of injected modes we set $\rho_{L(R)} = (\pi t)^{-1}$ were t is the nearest neighbor hopping of graphene.[S2]

The Hamiltonian of the central region (H_{TBG}) is described by a tight-binding model where the hopping amplitudes between sites i and j, $t_{ij}(d_{ij}) = V_{pp\sigma}(d_{ij})\cos^2(\phi) + V_{pp\pi}(d_{ij})\sin^2(\phi)$, where the bond length $d_{ij} = |\boldsymbol{d}_{ij}| = |\boldsymbol{R}_j - \boldsymbol{R}_i|$ and ϕ denotes the angle formed by $\boldsymbol{d}_{ij}$ and the z-axis. The value of the inter-atomic matrix elements is a function of the bond length:[S3,S4] $V_{pp\sigma} = V^0_{pp\sigma}e^{-\frac{d_{ij}-d_0}{\delta}}$, $V_{pp\pi} = V^0_{pp\pi}e^{-\frac{d_{ij}-a}{\delta}}$ where $V^0_{pp\sigma} = t^0_\perp = 0.48$ eV, $V^0_{pp\pi} = t_0 = -2.7$ eV, $a = 0.142$ nm, $d_0 = 0.335$ nm and $\delta = 0.184\sqrt{3}a$. To accurately describe the electronic properties of TBG, for each site i, the neighbours j are chosen inside a disc of radius $d_{ij} \leq 4a$. To include the effect of the in-plane magnetic field $\boldsymbol{B} = B\left(\cos\theta_B \boldsymbol{e}_x + \sin\theta_B \boldsymbol{e}_y\right)$ in the tight-binding Hamiltonian, we use the Peierls substitution where the hopping parameters are modified to $t_{ij} = t_{ij}e^{i\phi_{ij}}$ where $\phi_{ij} = ie\boldsymbol{A}\cdot\left(\boldsymbol{R}_i - \boldsymbol{R}_j\right)/\hbar$. In this expression the vector potential $\boldsymbol{A} = zB\left(\sin\theta_B \boldsymbol{e}_x - \cos\theta_B \boldsymbol{e}_y\right)$ is evaluated at $\left(\boldsymbol{R}_i + \boldsymbol{R}_j\right)/2$. Furthermore, we use a symmetric arrangement where top and bottom layer are located at $z = \pm d/2$ being $d = 0.335$ nm the interlayer distance.

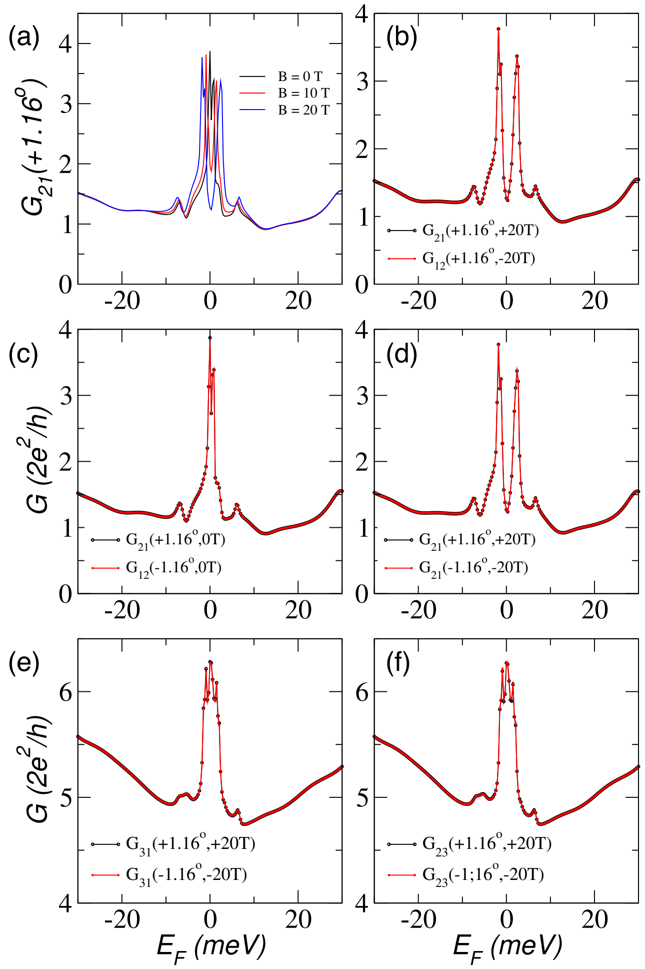

Figure S1: (Color online) Illustration of field dependence and symmetry properties of conductance matrix entries. (a) Conductance $G_{21}(\pm 1.16^{\circ}, B)$ in units of $2e^2/h$ as a function of the Fermi energy E_F, showing the effect of the in-plane magnetic field $\boldsymbol{B} = B\boldsymbol{e}_x$. (b) The reciprocity relations, $G_{21}(+\boldsymbol{B}) = G_{12}(-\boldsymbol{B})$. (c) $G_{21}(+\theta, \boldsymbol{B} = \boldsymbol{0}) = G_{21}(-\theta, \boldsymbol{B} = \boldsymbol{0})$. (d)-(f) The chiral reciprocity relations, $G_{pq}(\theta, \boldsymbol{B}) = G_{pq}(-\theta, -\boldsymbol{B})$.

Numerical validation of reciprocity relations

In the main text, we derived the standard and *chiral* reciprocity relation

$$G_{pq}(\theta, \boldsymbol{B}) = G_{qp}(\theta, -\boldsymbol{B}) \,, \tag{1}$$

$$G_{pq}(\theta, \boldsymbol{B}) = G_{pq}(-\theta, -\boldsymbol{B}) \,. \tag{2}$$

We will now numerically validate the reciprocity relations Eqs. (1) and (2) in order to show the reliability of our numerical approach. To this end, we distinguish the effect of an in-plane magnetic field on the conductance and then change twist angle and the field orientation. The black line in Fig. S1(a) corresponds to G_{21} for a TBG junction with $\theta = +1.16^\circ$ without magnetic field, a peak originated by Van Hove singularities around the CNP is clearly appreciated.[S5,S6] When the magnetic field is switched on along the $+x$-direction, the effect is mainly observed for low energies as a splitting of the conductance peak. This is produced by the separation of the Dirac points[S7–S9] as function of the strength and orientation of the field, that when projected onto the transport direction, appear at different energies (momentum) of the incoming electrons. Inverting the direction of the field, i.e., the field pointing to the negative x-direction, we observe that the traditional reciprocity relations, Eq. (1), are fulfilled. In Fig. S1(b), we only present the case $G_{21}(+1.16^\circ, +20\ T) = G_{12}(+1.16^\circ, -20\ T)$ to keep the discussion more transparent.

Without a magnetic field, it is not possible to detect the handedness of the TBG junction for layer-symmetric leads. To check this numerically, we again select the specific case $G_{21}(+1.16^\circ, 0\ T) = G_{12}(-1.16^\circ, 0\ T)$ in Fig. S1(c). However, this relation is no longer valid if an in-plane magnetic field is present as already anticipated. In Figs. S1(d)-(f), we recognize that the chiral reciprocity relations, Eq. (2), are fulfilled, i.e., $G_{21}(+1.16^\circ, +20\ T) = G_{21}(-1.16^\circ, -20\ T)$, $G_{31}(+1.16^\circ, +20\ T) = G_{31}(-1.16^\circ, -20\ T)$ and $G_{23}(+1.16^\circ, +20\ T) = G_{23}(-1.16^\circ, -20\ T)$. Although, in Fig. S1 we only focus on specific twist angles ($\theta = \pm 1.16^\circ$) and fields ($\boldsymbol{B} = \pm 20 \boldsymbol{e}_x\ T$) the reciprocity relations, Eqs. (1) and (2), are valid irrespective of the twist angle and field intensity.

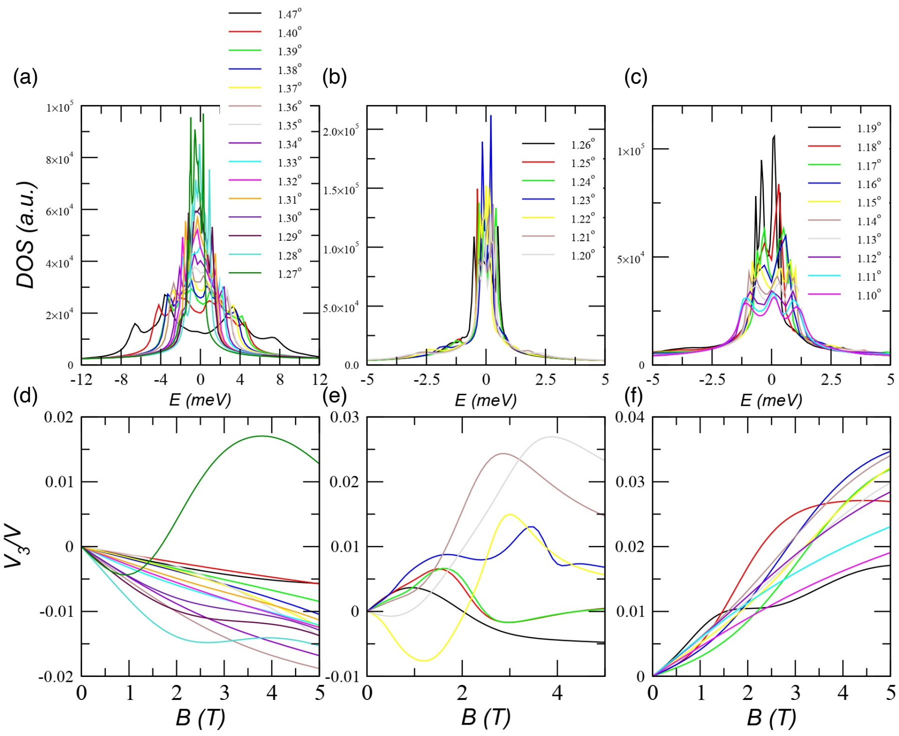

Figure S2: (Color online) (a)-(c) Density of states for different twist angles without magnetic field. (d)-(f) V_3/V for the angles indicated in the DOS.

Magic angle regime and density of states

In the main text, we presented the highest value and the full width half maximum (FWHM) of the density of states (DOS). Based on that, three regimes were identified and we labeled the second regime as magic angle or flat band regime as it displayed the largest peaks and the smallest FWHMs. The twist angles around 1.23° are slightly larger than the one found in experiments of $\sim 1.1°$ due to the finite system size considered here.

The DOS was calculated by summing the diagonal elements of the spectral function

$$\rho(E) = \frac{1}{2\pi}\mathrm{Tr}[A], \tag{3}$$

which is defined as

$$A = \mathcal{G}^R(\Gamma_1 + \Gamma_2 + \Gamma_3)\mathcal{G}^A. \tag{4}$$

The couplings $\Gamma_{1(2)(3)} = i[\Sigma_{1(2)(3)} - \Sigma^\dagger_{1(2)(3)}]$ and the retarded Green's function $\mathcal{G} = [E - H_{TBG} - \Sigma_1 - \Sigma_2 - \Sigma_3]^{-1}$ were defined in the main text. Note that we neglect the inclusion of an infinitesimal complex value in the retarded Green's to avoid any possible effect in the chiral response of the system.[S10] This fact has no effect on the transport properties because the self-energies (Σ_i) have their own imaginary part. However, they are non-zero only for sites in the vicinity of the leads. As a result, the peaks of the spectral function and consequently the DOS are not perfectly smoothed around the CNP where a large number of states is present.[S1]

In Fig. S2, we present the full DOS and also the voltage probe V_3 for a large range of twist angles between $\theta = 1.47° - 1.10°$. The left panel shows the results for twist angles $\theta > 1.26°$ where negative slopes in the linear regime are found for positive magnetic fields. The central panel presents the DOS and V_3 for twist angles around the magic angles ($1.20° \leq \theta \leq 1.26°$); in this regime, no definite slope can be assigned to the voltage probe V_3 around $B \approx 0$. Finally, the right panel shows the same information for twist angles $\theta < 1.20°$, where the slope is reversed and thus positive in the linear regime.

This result show that there is a change of chirality around the magic angle as found in the extended system.[S11] The observed voltage probe is thus an indication of an electronic chirality which is not directly linked with the real space chirality which does not change for positive twist angles.

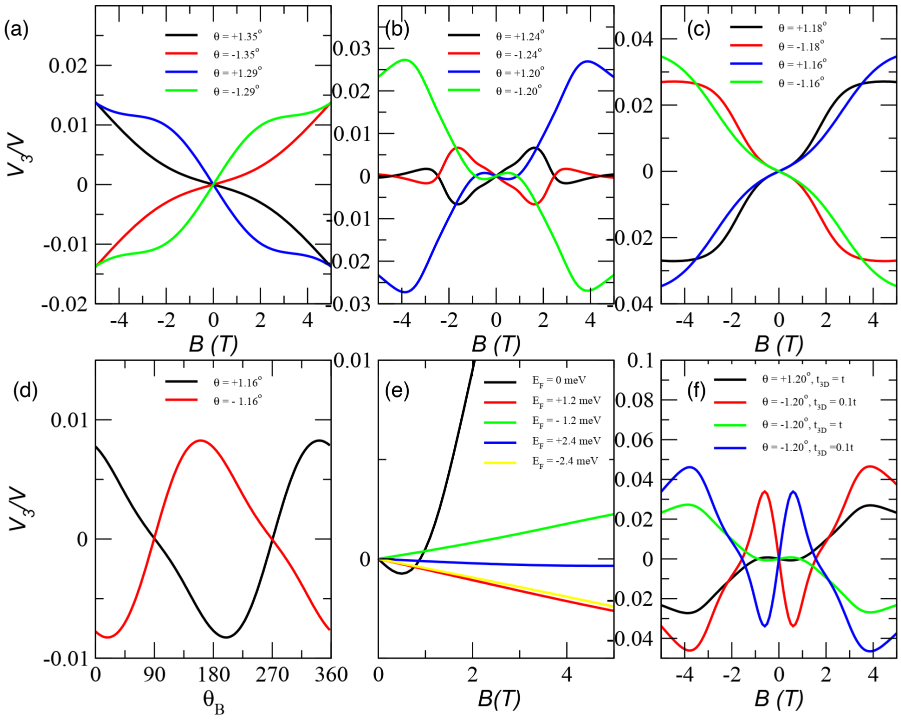

Figure S3: (Color online) (a)-(c) V_3 in units of V for the positive and negative twist angles with $E_F = 0$ and $\boldsymbol{B}$ parallel to the x-axis. (d) Angular dependence of V_3, where θ_B is the angle between the in-plane magnetic field and the x-axis, for $\theta = \pm 1.16°$ and $|\boldsymbol{B}| = 1$ T. (e) Effect of the Fermi energy on V_3 for $\theta = 1.20°$. (f) V_3 changing the coupling between the central region and lead 3 ($t_{3D} = 0.1t$).

Dependence of the voltage probe on magnetic field direction and coupling

Let us now discuss further results, i.e., the dependence of V_3 on negative magnetic fields, on the angle of the magnetic field with respect to the symmetry axis, on a backgate, as well as on the coupling to lead 3. In all cases we choose a symmetric source-drain voltage drop,$V_1 = -V_2 = V/2$.

Behavior for negative magnetic fields

The symmetry arguments of the main text indicate that the relation, $V_3(+\boldsymbol{B}, \theta) = -V_3(-\boldsymbol{B}, \theta)$, is valid to linear order in $\boldsymbol{B}$, in principle. Yet, the results seen in Figs. S3(a)-(c) point to an

exact relation. This is true because, in those Figs., the field is along the x direction of Fig. 1 of the main text, where an exact symmetry of our geometric setup holds, as explained later when considering non-linear effects. It is also obviously that the behavior is independent of whether the twist angle is above, around, or below the twist angle.

Angle-dependence of the magnetic field on the conductance

For the same reason, we have $V_3(+\theta, \boldsymbol{B}) = -V_3(-\theta, \boldsymbol{B})$ only in the linear field regime, in principle. As before if the magnetic field is along the x direction, this relation also holds in the non-linear regime. For arbitrary directions, however, this symmetry is slightly broken as seen in Fig. S3(d) that shows the angular dependence of V_3. In this Figure, θ_B denotes the angle between the in-plane magnetic field and the x-axis, for $\theta = \pm 1.16°$ and $|\boldsymbol{B}| = 1$ T. V_3 is thus not symmetric with respect to $\theta_B = \pi$ and the phenomenological correction term $\boldsymbol{I} \cdot \boldsymbol{B} = |\boldsymbol{I}||\boldsymbol{B}| \cos\theta_B$ that was discussed in a different context for macroscopic devices, see e.g. Refs. S12–S14, does not match our results.

Gate dependence

All results so far were obtained for a neutral twisted graphene sample. In Fig S3(e), we show results also for Fermi energies $E_F = \pm 1.2$ and $E_F = \pm 2.4$ meV. As can be seen, the linear regime becomes more evident for $\theta = 1.20°$ at finite gate voltage. For $E_F = \pm 1.2$meV, we also have $V_3(E_F) = -V_3(-E_F)$. This might have been expected as also the chiral part of the conductance is an odd function of the chemical potential.[S11]

However, this relation is not valid for $E_F = \pm 2.4$ meV. We believe this to be a finite size effect as also the DOS of the whole system as function of the twist angle changes, but more detailed simulations are needed in order to understand the gate dependence of the voltage probe.

Chiral engineering

Let us investigate the possibility of enhancing the chiral probe in the linear field regime as suggested by Eq. (9) of the main text. In Fig. S3(f), we show V_3 for $\theta = 1.20^\circ$ with a reduced coupling between the third lead and the central region ($t_{3D} = 0.1t$). As anticipated, the reduction of the coupling enhances the linear effect showing a possible route to engineer the chiral response.

Reciprocity relations for the two-terminal conductance

A dependence of the conductivity on the direction of a magnetic field is forbidden by the general Onsager relations. These state that for any linear response function $\mathcal{K}_{AB}$ one has $\mathcal{K}_{A,B}(\boldsymbol{B}) = \mathcal{K}_{\bar{B},\bar{A}}(-\boldsymbol{B})$ with A, B arbitrary operators and $\bar{A}, \bar{B}$ the time-reversed operators. For $A = B = j_x$, we have $\bar{A} = \bar{B} = -j_x$ and $\mathcal{K}_{j_x,j_x}(\boldsymbol{B}) = \mathcal{K}_{-j_x,-j_x}(-\boldsymbol{B}) = \mathcal{K}_{j_x,j_x}(-\boldsymbol{B})$.

This result also holds within the Landauer formalism in a two-terminal setup, because the unitarity of the S matrix imposes left-right reciprocity for any system attached to M_T modes, grouped into left and right leads, irrespective of any symmetry (or lack of) of the Hamiltonian. To show this, we first discuss the following relation for the S matrix:

$$\sum_{m=1}^{M_T} |s_{mn}|^2 = 1 \quad , \forall n \; , \tag{5}$$

see Datta (3.1.3b), where $m(n)$ run over all channels attached to our system. Using that $|s_{mn}|^2 = |s_{m\leftarrow n}|^2 = T_{m\leftarrow n}$ (Datta (3.1.1)) and grouping these channels into left(L) and right(R) leads, one has

$$\overline{T}_{LL} + \overline{T}_{R\leftarrow L} = N_L \; , \tag{6}$$

$$\overline{T}_{RR} + \overline{T}_{L\leftarrow R} = N_R \; . \tag{7}$$

where $N_{L(R)}$ is the number of channels in the $L(R)$ lead, with $N_R + N_L = M_T$, and

$$\overline{T}_{R\leftarrow L} = \sum_{m\in R}\sum_{n\in L} T_{m\leftarrow n} \ , \tag{8}$$

with similar relations for the other pairings, see Datta (3.1.2).

Unitarity also implies the "not so obvious relation" in Datta's own words, see Datta (3.1.3b) again,

$$\sum_{m=1}^{M_T} |s_{nm}|^2 = 1 \quad , \forall n \ , \tag{9}$$

which leads to

$$\overline{T}_{LL} + \overline{T}_{L\leftarrow R} = N_L \ , \tag{10}$$

$$\overline{T}_{RR} + \overline{T}_{R\leftarrow L} = N_R \ . \tag{11}$$

The combination of Eqs. (6) and (10) implies reciprocity:

$$\overline{T}_{R\leftarrow L} = \overline{T}_{L\leftarrow R} \ , \tag{12}$$

irrespective of any other consideration, except unitarity, always given in the non-dissipative, linear regime.

Relations beyond linear order

Let us finally discuss extensions to the linear approach presented in the main text. Quite generally, one can always write

$$\begin{bmatrix} I_1 \\ I_2 \\ I_3 \end{bmatrix} = (\boldsymbol{M}^s + \boldsymbol{M}^a) \begin{bmatrix} V_1 \\ V_2 \\ V_3 \end{bmatrix} , \tag{13}$$

where $\boldsymbol{M}^s$ is a symmetric matrix, *even* in chirality and field, which we can denote as $\boldsymbol{M}^s(\theta^2, \boldsymbol{B}^2)$. $\boldsymbol{M}^a$ is an asymmetric matrix that, from unitarity, depends on a single entry

$$\boldsymbol{M}^a = \begin{bmatrix} 0 & -\delta G & +\delta G \\ +\delta G & 0 & -\delta G \\ -\delta G & +\delta G & 0 \end{bmatrix} , \tag{14}$$

with

$$\delta G = \frac{G_{12} - G_{21}}{2} = -\frac{G_{13} - G_{31}}{2} = \frac{G_{23} - G_{32}}{2}, \tag{15}$$

where now, δG is *odd* in chirality and field, which we can describe as

$$\delta G(\theta, \boldsymbol{B}) = \sigma\sigma' \delta G(\sigma\theta, \sigma' \boldsymbol{B}), \tag{16}$$

with $\sigma(\sigma') = \pm 1$. In the main text, we restricted δG to lowest (linear) order in $\boldsymbol{B}$, but it can be any odd function of field and chirality, generalizing Eq. (9) of the main text.

The voltage probe in the case of $I_3 = 0$ can be written as

$$V_3 = \frac{V_1 + V_2}{2} + \delta\tilde{V_3} \frac{V_1 - V_2}{2}, \tag{17}$$

with

$$\delta\tilde{V_3} = \delta\tilde{V_3}^e + \delta\tilde{V_3}^o, \tag{18}$$

where $\delta\tilde{V}^e = \delta\tilde{V}^e(\theta^2, \boldsymbol{B}^2)$ is an even function of field and chirality, given explicitly by

$$\delta\tilde{V}_3^e = \frac{(G_{31}+G_{13})-(G_{32}+G_{23})}{2(G_{31}+G_{32})} \,. \tag{19}$$

It vanishes for $\boldsymbol{B}=0$ in our left-right symmetrically chosen attachment for the third lead as $G_{31}=G_{32}$.

On the other hand, $\delta\tilde{V}^o$, with expression

$$\delta\tilde{V}_3^o = \frac{(G_{31}-G_{13})-(G_{32}-G_{23})}{2(G_{31}+G_{32})} = \frac{\delta G}{(G_{31}+G_{32})}, \tag{20}$$

is an odd function of field and chirality, formally:

$$\delta\tilde{V}^o(\theta,\boldsymbol{B}) = \sigma\sigma'\tilde{V}^o(\sigma\theta,\sigma'\boldsymbol{B}), \tag{21}$$

with $\sigma(\sigma')=\pm 1$. Notice that to linear order in $\boldsymbol{B}$, one has $\delta\tilde{V}_3(\theta,\boldsymbol{B}) = -\delta\tilde{V}_3(-\theta,\boldsymbol{B})$, as asserted in the main text. This relation needs not hold beyond linear order, but, nevertheless, this does not compromise the chiral sensitivity of the voltage probe for, in general, $\delta\tilde{V}_3(\theta,\boldsymbol{B}) \neq \delta\tilde{V}_3(-\theta,\boldsymbol{B})$.

In spite of the above generalizations, our numerical calculations show that, when the field is in the x-direction, one always has

$$\delta\tilde{V}_3(\theta,B) = -\delta\tilde{V}_3(-\theta,B), \quad \boldsymbol{B}=B\boldsymbol{e}_x \,, \tag{22}$$

as seen e.g. in Fig. 2 a,b,c. This is no accident, because in our geometry, a π rotation around the y-axis reverses B and exchanges leads 1 and 2, leading necessarily to Eq. (22) as an exact statement - beyond linear order. This symmetry is also responsible for the vanishing of $\delta\tilde{V}_3$ when the field is oriented along the y-direction, see Fig. 2 d.

As said, Eq. (22) no longer holds beyond linear order when the field is oriented in an

arbitrary direction, as illustrated in Fig. 2 d. Nevertheless, non-linear effects are rather small, at least for $B = 1T$, as can be seen in that figure. Moreover, as previously asserted, the chiral sensitivity of the probe remains such that $\delta\tilde{V}_3(\theta, \boldsymbol{B}) \neq \delta\tilde{V}_3(-\theta, \boldsymbol{B})$.

Landauer-Büttiker description of the layer-discriminating setup without magnetic field

Now we have four leads: 1, 2, 3t, and 3b. Therefore,

$$\begin{pmatrix} I_1 \\ I_2 \\ I_{3t} \\ I_{3b} \end{pmatrix} = \begin{pmatrix} G_{21}+G_{3t1}+G_{3b1} & -G_{21} & -G_{3t1} & -G_{3b1} \\ -G_{21} & G_{21}+G_{3t1}+G_{3b1} & -G_{3b1} & -G_{3t1} \\ -G_{3t1} & -G_{3b1} & G_{3b3t}+G_{3t1}+G_{3b1} & -G_{3b3t} \\ -G_{3b1} & -G_{3t1} & -G_{3b3t} & G_{3b3t}+G_{3t1}+G_{3b1} \end{pmatrix} \begin{pmatrix} V_1 \\ V_2 \\ V_{3t} \\ V_{3b} \end{pmatrix}, \tag{23}$$

where we have made use of the reciprocity in the absence of magnetic field, $G_{ij} = G_{ji}$, and the additional symmetry of our geometric arrangement, $G_{3t\,2} = G_{3b1}$ and $G_{3t1} = G_{3b2}$.

Detecting chirality with transverse currents: layer contrasting Hall effect

If the terminals $3t$ and $3b$ are kept at the same potential, $V_{3t} = V_{3b}$, then

$$\frac{I_{3b} - I_{3t}}{V_1 - V_2} = G_{3t1} - G_{3b1}, \tag{24}$$

as asserted in the main text, with a finite value of this magnitude implying chirality.

For $V_1 = V/2$, $V_2 = -V/2$, and $V_{3b} = V_{3t} = 0$, in addition to the standard source-drain current between 1 and 2 reservoirs, one gets transverse currents,

$$I_{3b} + I_{3t} = 0, \tag{25}$$

and, therefore, no net current flows from the system to reservoirs $3t + 3b$, kept at the same potential $V_3 = 0$. Yet a finite, $|I_{3t}| = |I_{3b}|$, and layer-opposite current in the transverse leads emerges due to chirality, as depicted in Fig. 3 (a) and (b) of the main text.

The experimental detection of these transverse currents could be problematic, for real leads are always resistive. Nevertheless, the same physics can be exposed with an alternative measurement, where the transverse leads are used as independent voltage probes, and chirality manifests as different voltage readings, $V_{3t} \neq V_{3b}$. This voltage difference can be interpreted physically as the chemical potential difference due to the carrier accumulation-depletion of the "frustrated supercurrent" at the edges. The new conditions would be $V_1 = V/2$, $V_2 = -V/2$, and $I_{3b} = I_{3t} = 0$. Eq. 23 then gives

$$V_{3t} - V_{3b} = \frac{G_{3t1} - G_{3b1}}{G_{3t1} + G_{3b1} + 2G_{3b3t}} V. \tag{26}$$

Notice that the denominator of Eq. 26 is even in chirality and, therefore, a finite value of $(G_{3t\,1} - G_{3b\,1})$, opposite for opposite chiralities $(\pm\theta)$, reveals the chirality, as in the case of current detection.

Complementary setup for chirality detection

Guided by results for the conductivity in an infinite system,[S15,S16] in the main text, we discussed a complementary setup based on the same symmetry properties. It consists of forcing the opposite transverse currents by means of a voltage drop in the corresponding reservoirs, $V_{3t} = +V/2$, $V_{3b} = -V/2$, and then observing as chiral probe the emergence of a net current between reservoirs 1 and 2, without any voltage drop between the terminals, $V_1 = V_2 = 0$, a "supercurrent" in some sense, see Fig. 3 (c) - (d) of main text. Under the specified conditions, $V_{3t} = +V/2$, $V_{3b} = -V/2$, $V_1 = 0$, $V_2 = 0$, Eq. 23 leads to

$$I_1 = -I_2 = (G_{3b\,1} - G_{3t\,1})V/2, \tag{27}$$

that is, the appearance of a net longitudinal current between reservoirs 1 and 2. To avoid potential misunderstandings, notice that the minus sign of I_2 in Eq. 27 comes from usual convention in the Büttiker formalism that a positive I_i means current *injected* from reservoir i to the system, and viceversa. Therefore, Eq. 27 describes the same amount of current entering to the flake from reservoir 1 and exiting to reservoir 2, again, without any voltage drop between them, $V_1 = V_2 = 0$.

As in the original arrangement of the manuscript, the effect requires chirality manifested as a finite value of $(G_{3t\,1} - G_{3b\,1})$, opposite for opposite chiralities $(\pm\theta)$. The value of $(G_{3t\,1} - G_{3b\,1})$ as function of Fermi level in twisted bilayer graphene was plotted in the main manuscript.

As before, experimental detection of this "supercurrent" with real resistive leads can be problematic. We can then opt for using leads 1 and 2 as voltage probes, as in the previous case. The new conditions would be $V_{3t} = +V/2$, $V_{3b} = -V/2$, and $I_1 = I_2 = 0$. Eq. 23 then gives

$$V_1 - V_2 = \frac{G_{3t1} - G_{3b1}}{G_{3t1} + G_{3b1}} V. \tag{28}$$

As in Eq. 26, the denominator of Eq. 28 is even in chirality and, once again, it is the finite value of $(G_{3t\,1} - G_{3b\,1})$, opposite for opposite chiralities $(\pm\theta)$, what reveals the chirality.

Change in chirality in the infinite system with time-reversal symmetry

In Ref. S11, it was shown that electronic chirality is a Fermi-surface property and given by the chiral Drude weight

$$D_{xy} = \frac{1}{2A} \sum_{\boldsymbol{k},n} \boldsymbol{e}_z \cdot (\boldsymbol{j}^1_{\boldsymbol{k},n} \times \boldsymbol{j}^2_{\boldsymbol{k},n}) \delta(\epsilon_{\boldsymbol{k},n} - E_F) \,. \tag{29}$$

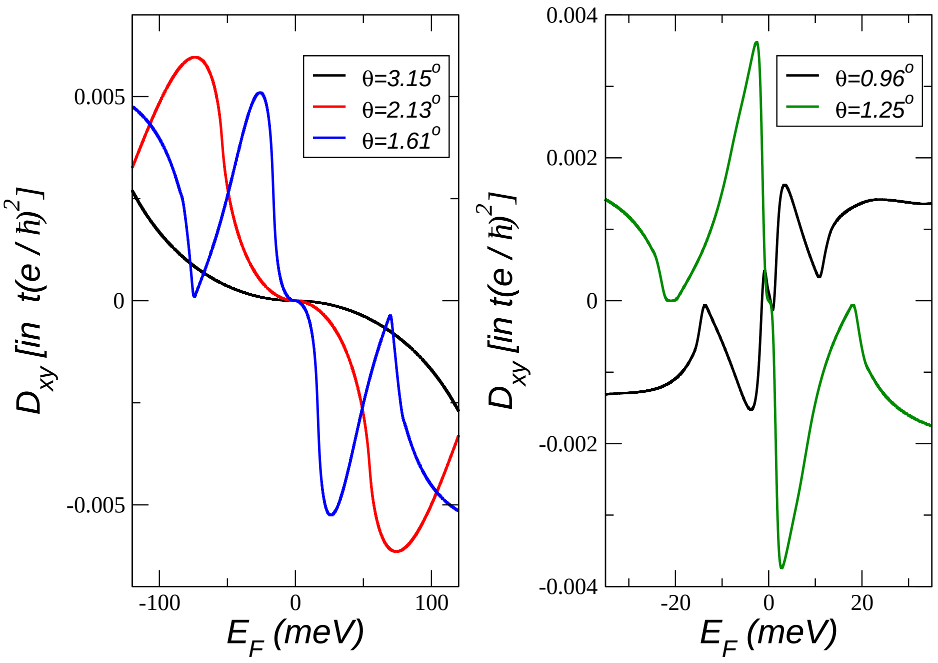

Figure S4: (Color online) Left panel: The chiral Drude weight for large twist angles. Right panel: The chiral Drude weight below and above the magic angle $\theta_m = 1.08°$.

It is defined by the sheet currents of layer ℓ denoted by $\boldsymbol{j}^{\ell}_{\boldsymbol{k},n}$ and sum over all states labeled by the band index n and Bloch states $\boldsymbol{k}$ with energies around the Fermi energy E_F. Furthermore, A denotes the area of the sample and the eigenenergies $\epsilon_{\boldsymbol{k},n}$ are given in units of the carbon-carbon hopping amplitude $t \sim 2.7$eV.

The continuum model of twisted bilayer graphene shows an approximate particle-hole symmetry. Then the chiral Drude weight is in an odd function of the Fermi energy with $D_{xy}(E_F) = -D_{xy}(-E_F)$. The change in chirality at the neutrality point can be seen on the left and right panel of Fig. S4. However, for large angles the chirality is always positive for $E_F < 0$. This changes when the twist angle is below the magic angle $\theta_m = 1.08°$ and the chirality for e.g. $\theta = 0.96°$ is negative for $E_F < 0$.

Interestingly, the shape of the curves is similar to the one of the right panel for positive twist angle of Fig. 4 of the main text. In contrary to the case of the infinite system, these

curves do not change around zero due to a residual chirality which is due to finite size effects and not related to the band structure.

References

(S1) Datta, S. *Electronic Transport in Mesoscopic Systems*; Cambridge University Press, 1995.

(S2) Bahamon, D. A.; Gómez-Santos, G.; Stauber, T. Emergent magnetic texture in driven twisted bilayer graphene. *Nanoscale* **2020**, *12*, 15383–15392.

(S3) Brihuega, I.; Mallet, P.; González-Herrero, H.; Trambly de Laissardière, G.; Ugeda, M. M.; Magaud, L.; Gómez-Rodríguez, J. M.; Ynduráin, F.; Veuillen, J.-Y. Unraveling the Intrinsic and Robust Nature of van Hove Singularities in Twisted Bilayer Graphene by Scanning Tunneling Microscopy and Theoretical Analysis. *Phys. Rev. Lett.* **2012**, *109*, 196802.

(S4) Moon, P.; Koshino, M. Energy spectrum and quantum Hall effect in twisted bilayer graphene. *Phys. Rev. B* **2012**, *85*, 195458.

(S5) de Castro, S. G.; Ferreira, A.; Bahamon, D. A. Efficient Chebyshev polynomial approach to quantum conductance calculations: Application to twisted bilayer graphene. *Phys. Rev. B* **2023**, *107*, 045418.

(S6) de Castro, S. G.; Lopes, J. a. M. V. P.; Ferreira, A.; Bahamon, D. A. Fast Fourier-Chebyshev Approach to Real-Space Simulations of the Kubo Formula. *Phys. Rev. Lett.* **2024**, *132*, 076302.

(S7) Kwan, Y. H.; Parameswaran, S.; Sondhi, S. Twisted bilayer graphene in a parallel magnetic field. *Physical Review B* **2020**, *101*, 205116.

(S8) Pershoguba, S. S.; Yakovenko, V. M. Energy spectrum of graphene multilayers in a parallel magnetic field. *Physical Review B* **2010**, *82*, 205408.

(S9) De Gail, R.; Fuchs, J.-N.; Goerbig, M.; Piéchon, F.; Montambaux, G. Manipulation of Dirac points in graphene-like crystals. *Physica B: Condensed Matter* **2012**, *407*, 1948–1952.

(S10) Liu, Y.; Holder, T.; Yan, B. Chirality-induced giant unidirectional magnetoresistance in twisted bilayer graphene. *The Innovation* **2021**, *2*, 100085.

(S11) Stauber, T.; González, J.; Gómez-Santos, G. Change of chirality at magic angles of twisted bilayer graphene. *Phys. Rev. B* **2020**, *102*, 081404.

(S12) Rikken, G. L. J. A.; Fölling, J.; Wyder, P. Electrical Magnetochiral Anisotropy. *Phys. Rev. Lett.* **2001**, *87*, 236602.

(S13) Wagnière, G. H. *On chirality and the universal asymmetry: reflections on image and mirror image*; John Wiley & Sons, 2007.

(S14) Tokura, Y.; Nagaosa, N. Nonreciprocal responses from non-centrosymmetric quantum materials. *Nature Communications* **2018**, *9*, 3740.

(S15) Stauber, T.; Low, T.; Gómez-Santos, G. Chiral Response of Twisted Bilayer Graphene. *Phys. Rev. Lett.* **2018**, *120*, 046801.

(S16) Stauber, T.; Low, T.; Gómez-Santos, G. Linear response of twisted bilayer graphene: Continuum versus tight-binding models. *Phys. Rev. B* **2018**, *98*, 195414.
